# Long-term neurocognitive dysfunction in offspring via NGF/ ERK/CREB signaling pathway caused by ketamine exposure during the second trimester of pregnancy in rats

**DOI:** 10.18632/oncotarget.16042

**Published:** 2017-03-09

**Authors:** Yanan Li, Xinran Li, Cen Guo, Lina Li, Yuxin Wang, Yiming Zhang, Yu Chen, Wenhan Liu, Li Gao

**Affiliations:** ^1^ College of Veterinary Medicine, Northeast Agricultural University, Harbin 150030, China

**Keywords:** ketamine, pregnancy, long-term neurocognitive dysfunction, ERK, synapse

## Abstract

Early life exposure to ketamine caused neurohistopathologic changes and persistent cognitive dysfunction. For this study, a pregnant rat model was developed to investigate neurocognitive effects in the offspring, following ketamine exposure during the second trimester. Pregnant rats on gestational day 14 (equal to midtrimester pregnancy in humans), intravenously received 200 mg/kg ketamine for 3 h. Their behavior was tested (Morris water maze, odor recognition test, and fear conditioning) at postnatal days (P25–30). Furthermore, hippocampal morphology of the offspring (P30) was examined via Nissl staining and hippocampal dendritic spine density was determined via Golgi staining. The hippocampal protein levels of nerve growth factor (NGF), extracellular signal-regulated kinase (ERK), phosphorylated-ERK (p-ERK), cyclic adenosine monophosphate response element-binding (CREB), p-CREB, synaptophysin (SYP), synapsin (SYN), and postsynaptic density-95 (PSD95) were measured via western blot. Additionally, SCH772984 (an ERK inhibitor) was used to evaluate both role and underlying mechanism of the ERK pathway in PC12 cells. We found that ketamine caused long-term neurocognitive dysfunction, reduced the density of the dendritic spin, caused neuronal loss, and down-regulated the expression of NGF, ERK, p-ERK, mitogen, and stress-activated protein kinase (MSK), CREB, p-CREB, SYP, SYN, and PSD95 in the hippocampus. These results suggest that ketamine induced maternal anesthesia during period of the fetal brain development can cause long-term neurocognitive dysfunction in the offspring, which likely happens via inhibition of the NGF-ERK-CREB pathway in the hippocampus. Our results highlight the central role of ERK in neurocognition.

## INTRODUCTION

A large number of pregnant women are exposed to various types of anesthetics for surgery or diagnosis every year. Furthermore, some pregnant women will also accept surgery unrelated to childbirth during pregnancy. Published evidence links early exposure to anesthesia and cognitive impairment in the offspring [1, 2]. For example, exposure to isoflurane during pregnancy can impair postnatal spatial memory and learning in the offspring [3]. Moreover, neonatal administration of commonly used anesthetic agents has been shown to induce long-term neurocognitive dysfunction in rodents [4]. Ketamine is one of the most commonly used drugs in pediatric clinical anesthesia. Its influence on learning and memory has always been of clinical concern. Ketamine is frequently consumed by drug addicts in the public, sadly including pregnant women [5]. It has been reported that ketamine had neurotoxic effects on brain development in rodents and even in primates, which persist into adulthood [6]. Ketamine may interfere with hippocampal neurogenesis and long-term neurocognitive function in rats up to seven days after birth [7]. These findings raise concerns about the potential adverse effects of ketamine exposure for fetuses and infants.

It is well known that the hippocampus plays a central role in processes of learning and memory [8], and it is therefore a common target for drug regulation. Synaptic plasticity refers to the change of neurotransmitter transmissions between two neurons or synapses and this is often accompanied by changes in synaptic morphology and function [9]. It is generally accepted that the hippocampal neuron synapses are associated with learning and memory [10]. Furthermore, synaptic plasticity has been suggested as the main cellular mechanism of memory [11], including synaptic formation and changes in synaptic associated protein [12]. Analysis of the synapse associated protein was coupled with that of synaptic markers such as synaptophysin (SYP), synapsin (SYN), and postsynaptic density-95 (PSD95). Among these, SYP and SYN are major integral proteins of synaptic vesicles, and their reduction has been associated with a decrease in synaptic density [13]; furthermore, PSD95 is used as a marker of dendrite branching and remodeling [14]. In addition, the nerve growth factor (NGF) plays a central role in promoting neurite outgrowth, thus enhancing memory formation in the hippocampus [15]. Chronic intracerebroventricular injection of NGF has been shown to increase the number of synapses in the hippocampus of rats, via up-regulation of SYP expression, leading to enhanced cognitive function [16]. Extracellular signal-regulated kinase (ERK) is a member of the mitogen-activated protein kinase (MAPK) pathway. ERK signaling is also involved in gene transcription, and it can induce and maintain memory [17]. Mitogen and stress-activated protein kinase (MSK) is a potent cyclic-AMP response element binding protein (CREB) kinase and phosphorylated CREB can initiate transcription of its target genes. Furthermore, CREB, the downstream regulator of the ERK cascade, has also been reported to be involved in the development of learning and memory and it has been described as an essential factor for increasing the number of synapses [18]. The activation of the ERK-CREB signaling pathway may improve spatial learning and memory [19]. These findings suggest that the proteins mentioned above are closely related to long-term neurocognition.

However, most of the existing studies in this area have focused on babies rather than offspring and the underlying mechanism remain unclear. Although ketamine is rarely administered to pregnant women in the developed countries, it is still often used as a “rescue” anesthetic when local anesthesia cannot meet normal surgical requirements in developing countries [20]. Therefore, it is important to reveal whether ketamine use during surgery or ketamine abuse during pregnancy poses a risk to unborn babies. The present study used a pregnant rat model and ketamine was injected into pregnant rats after 14 days of gestation (G14). We evaluated the capacity of learning and memory with behavioral tests (Morris water maze, odor recognition test, and fear conditioning), Nissl staining, and Golgi staining. The hippocampal protein level of NGF, ERK, p-ERK, CREB, p-CREB, SYP, SYN, and PSD95 were measured via western blot. Furthermore, *in vitro* cultured PC12 cells were used to validate the results. The aim of our study was to investigate the effects of ketamine exposure during the second trimester on neurocognition of the offspring.

## RESULTS

### Physiologic response to ketamine infusion

All anesthetized dams recovered fully without any complications. The recovery time from anesthesia was 36 ± 3 min. Maternal blood gas, blood glucose, and body temperature measurements were within the normal physiological range compared with control rats (Table 1). The pups weight was 83 ± 5 g. Two offspring had extremely low body weight (38 g and 45 g) and were excluded from further studies.

**Table 1:** Blood gases, glucose and temperature measurements in age-matched pregnant and unpregnant rats during ketamine administration

Arterial blood gas	Unpregnancy	Pregnancy
Temperature (°C)	36.89 ± 0.35	37.11 ± 0.50
PaO2 (mm Hg)	103.73 ± 3.08	104.17 ± 3.44
PaCO2 (mm Hg)	39.54 ± 1.56	42.98 ± 2.92
HCO3−	23.90 ± 1.23	24.60 ± 1.71
pH	7.18 ± 0.01	7.23 ± 0.02
Glucose (mg/dl)	103.73 ± 1.76	107.97 ± 2.69

Arterial blood samples were sampled from the left cardiac ventricle immediately at the end of the 3 h ketamine administration. All measurements were within the normal physiological ranges and no statistical significance was found between the two groups. Data are presented as mean ± S.E.M. (*n* = 3).

### Hippocampal-dependent learning and memory of offspring after ketamine exposure

#### Morris water maze task

**Figure 1:**
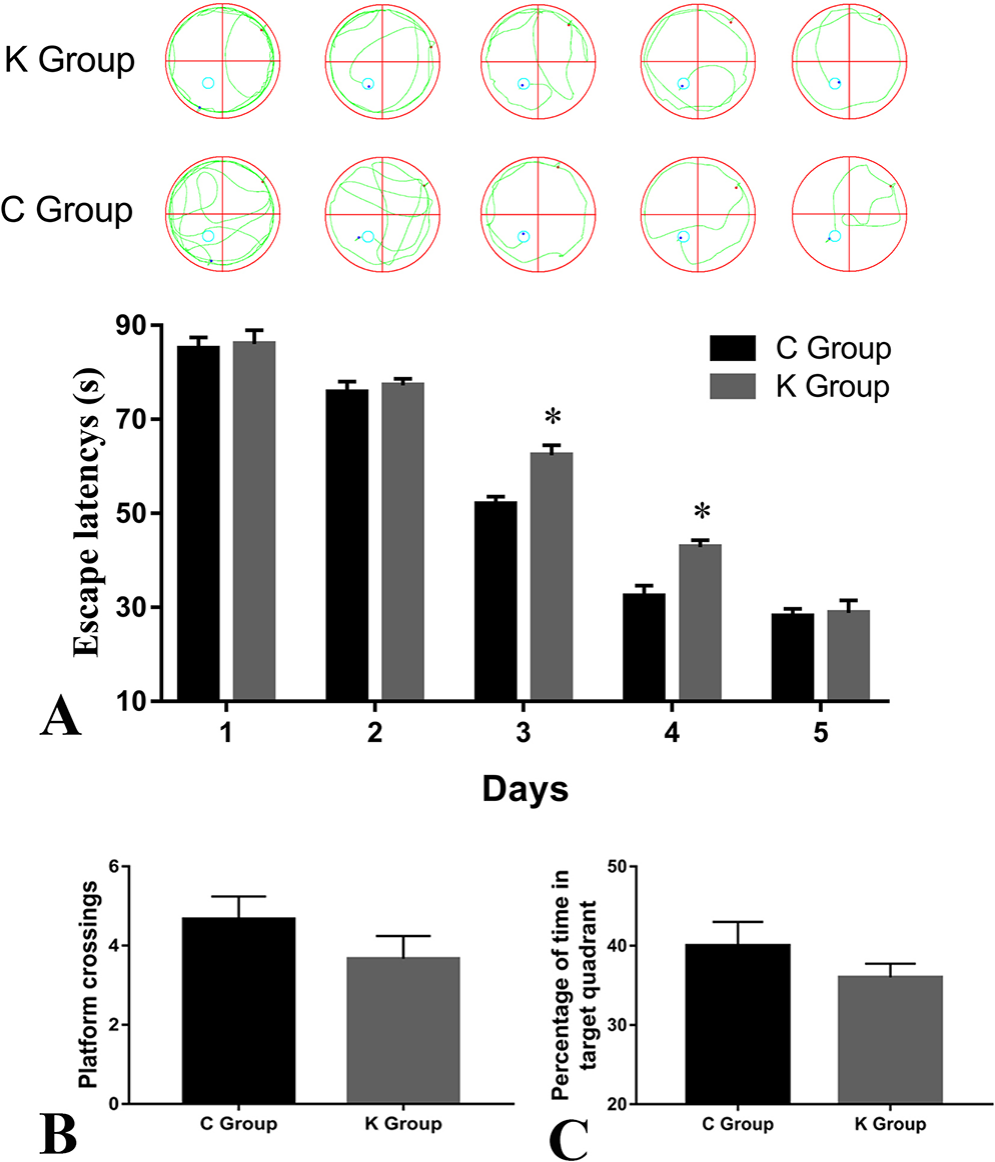
Morris water maze task Anesthesia-induced spatial cognitive changes in postnatal day 30 rats. Representative swimming paths of rats are shown above the A **(A)** Escape latency was significantly longer in the K group compared to the C group. **(B)** The number of platform crossings did not significantly differ between K and C groups. **(C)** The percentage of time in target quadrant did not significantly differ between K and C groups. *p < 0.05 *n* = 15.

The effects of ketamine exposure on spatial learning and memory in rats are shown in Figure 1. The Morris water maze test data in Figure 1A showed no significant differences in escape latencies between C group and K group during test days 1–3. However, on the third and forth day, escape latencies of the K group were significantly longer compared to C group (*p* < 0.05). On the fifth day, the data showed that the effect of ketamine exposure on the escape latency did not significantly differ between both groups. This suggested that both groups of rats were successfully trained after all. In the spatial probe test data, as shown in Figure 1B and Figure 1C, the number of platform crossings and the time of entries into the quadrant zone were shorter in K groups compared to C group. These findings indicate that ketamine-treated rats have a weaker spatial memory than control rats. Ketamine may thus impair spatial learning and memory ability of new-born rats.

### Odor recognition test

All rats in our experiments had never before been exposed to the utilized compounds and did not show a preference for either of both odors at the concentrations used in this study. During the acquisition test (as shown in Figure 3B), the animals spent considerable time investigating each of the two odorized holes and no significant difference could be detected between groups. The data of the recall test showed that the rats spent almost the same amount of time exploring each hole, indicating that they no longer remembered the odor presented during the acquisition test and thus, both odors were considered as unfamiliar in the recall test. Therefore, these findings indicate that ketamine had little effect on olfactory memory.

**Figure 2:**
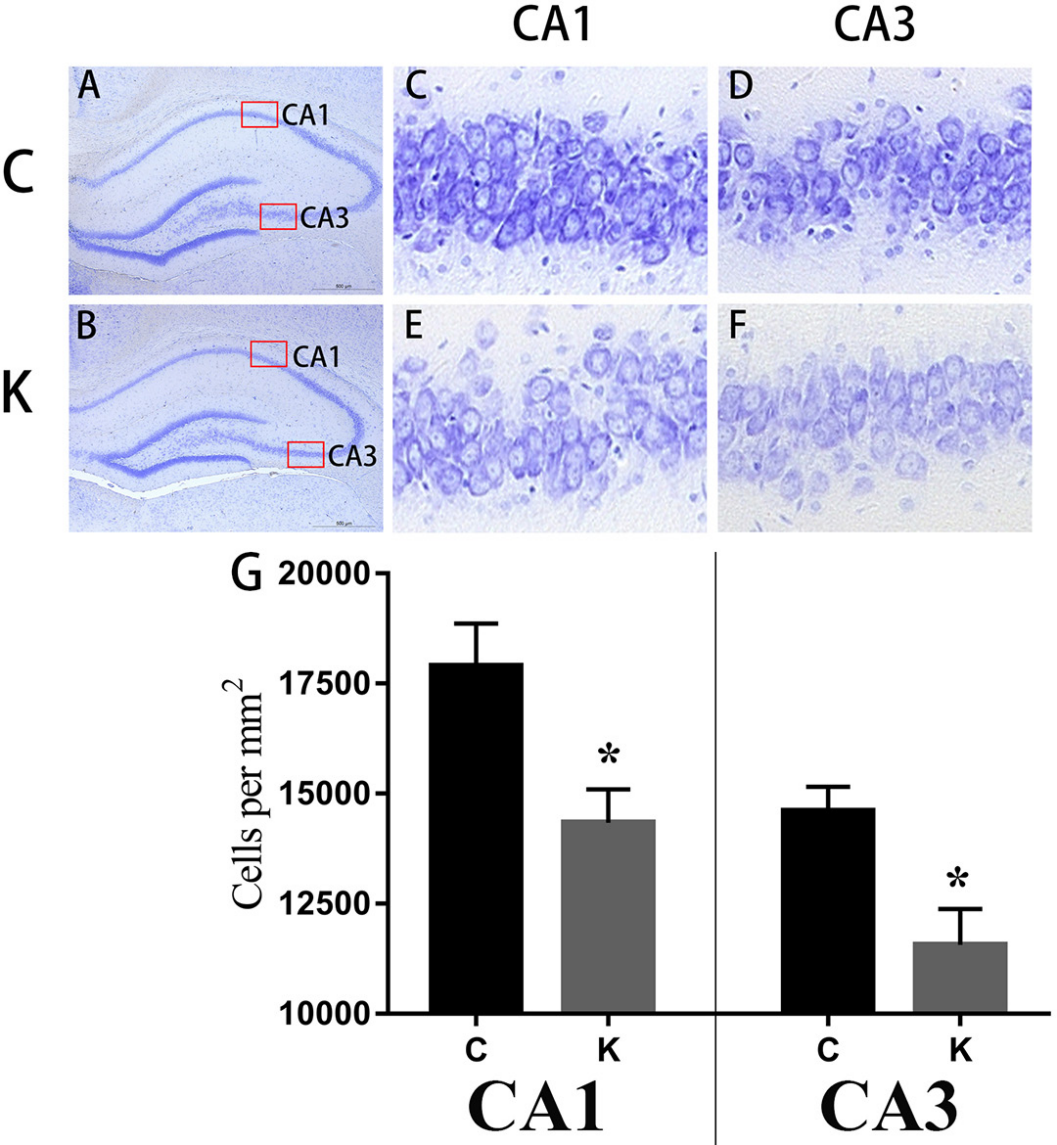
Nissl staining Ketamine administered during the second trimester induced neuronal cell loss in the hippocampal CA1 and CA3 regions of offspring. Coronal sections of the dorsal hippocampus from six animals were randomly selected from each group (*n* = 3/group) at postnatal day 30 (after behavioral tests) for Nissl-staining. Gross architecture of the hippocampus in the control and ketamine treated offspring at day 30 **(A** and **B**, respectively), the cellular layer in the CA1 and CA3 region of hippocampi of control and ketamine treated offspring at day 30 **(C–F**), from which cell densities were calculated. Significant differences were found in cell density in CA1 and CA3 between both groups. *p < 0.05.

**Figure 3:**
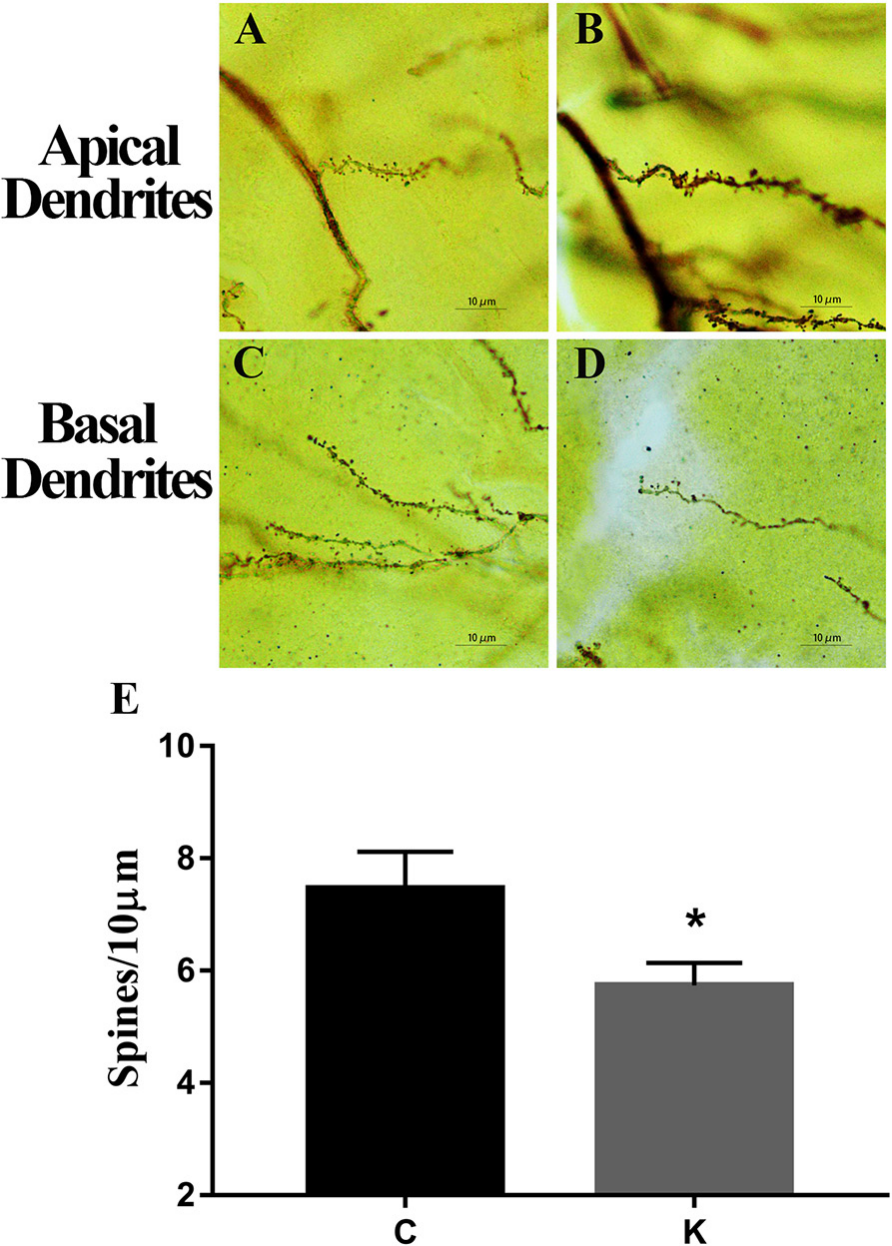
Golgi staining Ketamine decreased the spinal density of pyramidal neurons from the hippocampus. Ten apical and basal dendritic sections were measured from each rat (5 rats/group). Analysis revealed that ketamine treatment induced a significant reduction in spinal density. **(A–D)** Representative spinal morphology of the hippocampus in two groups. The histogram shows mean ± SEM of the dendritic spine numbers per 10 μm of dendrite length. **(E)** There were significant differences between K and C groups. *p < 0.05.

### Fear conditioning

**Figure 4:**
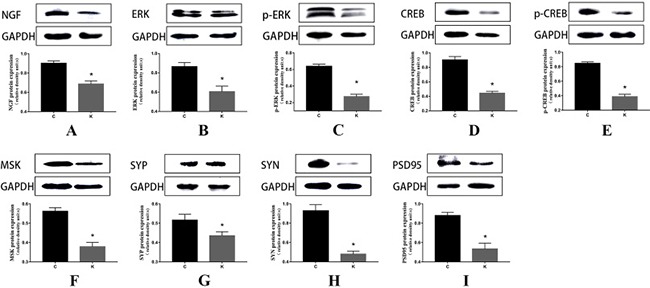
Western blot *in vivo* experiments Ketamine administered during the second trimester perturbed NGF and down-regulated ERK, p-ERK, MSK, CREB, p-CREB, SYP, SYN, and PSD95 in the hippocampi of offspring. On postnatal day 30, after behavioral tests, brains were harvested for western blot analyses to determine the expression of NGF, ERK, p-ERK, MSK, CREB, p-CREB, SYP, SYN, and PSD95. **(A–I**) Above are representative immunoblots for the expression levels of the corresponding proteins, and below are the histograms of the quantification of the corresponding proteins normalized to GAPDH. Data are expressed as mean percentage ± SEM of control mean values. *p < 0.05.

In both context test and pre-CS test, the percentage of freezing time between both groups was not significantly different (Figure 4D). While there were significant differences (*p* < 0.05) between both groups in the CS test, the percentage of freezing time was higher in C group than in K group (Figure 4D). This indicates that ketamine affects learn and memory of rats under conditioning stimuli.

### Ketamine induced neuronal cell loss in the hippocampal CA1 and CA3 region of offspring

The hippocampus is highly connected to learning and memory as well as mood associated behaviors [21]. Nissl staining was used to distinguish viable neurons from apoptotic or neurotic neurons. The former exhibited abundant cytoplasm and Nissl substance (stained as dark blue), apparent oval nuclei (stained as light blue), and prominent nucleoli, while apoptotic or necrotic cells exhibited pyknotic morphology with amorphous or fragmented nuclei. Significant differences (p < 0.05) were detected in cell density in the CA1 and CA3 region between C group and K group (Figure 2).

### Ketamine decreases dendritic spine density in offspring

Fully impregnated CA1 and CA3 pyramidal cells were detected under Golgi staining, and the spines of the apical and basal dendrites (Figure 3A–3D) were analyzed under a light microscope with 100× oil immersion objective lens. Merely the density of the dendritic spine was determined in our study, as the different types of spines were not always clearly distinguishable (e.g., thin, mushroom, or branched dendrites). The spine density of K group was significantly decreased compared to that of C group (*p* < 0.05).

### Ketamine exposure affects synaptic protein expression

**Figure 5:**
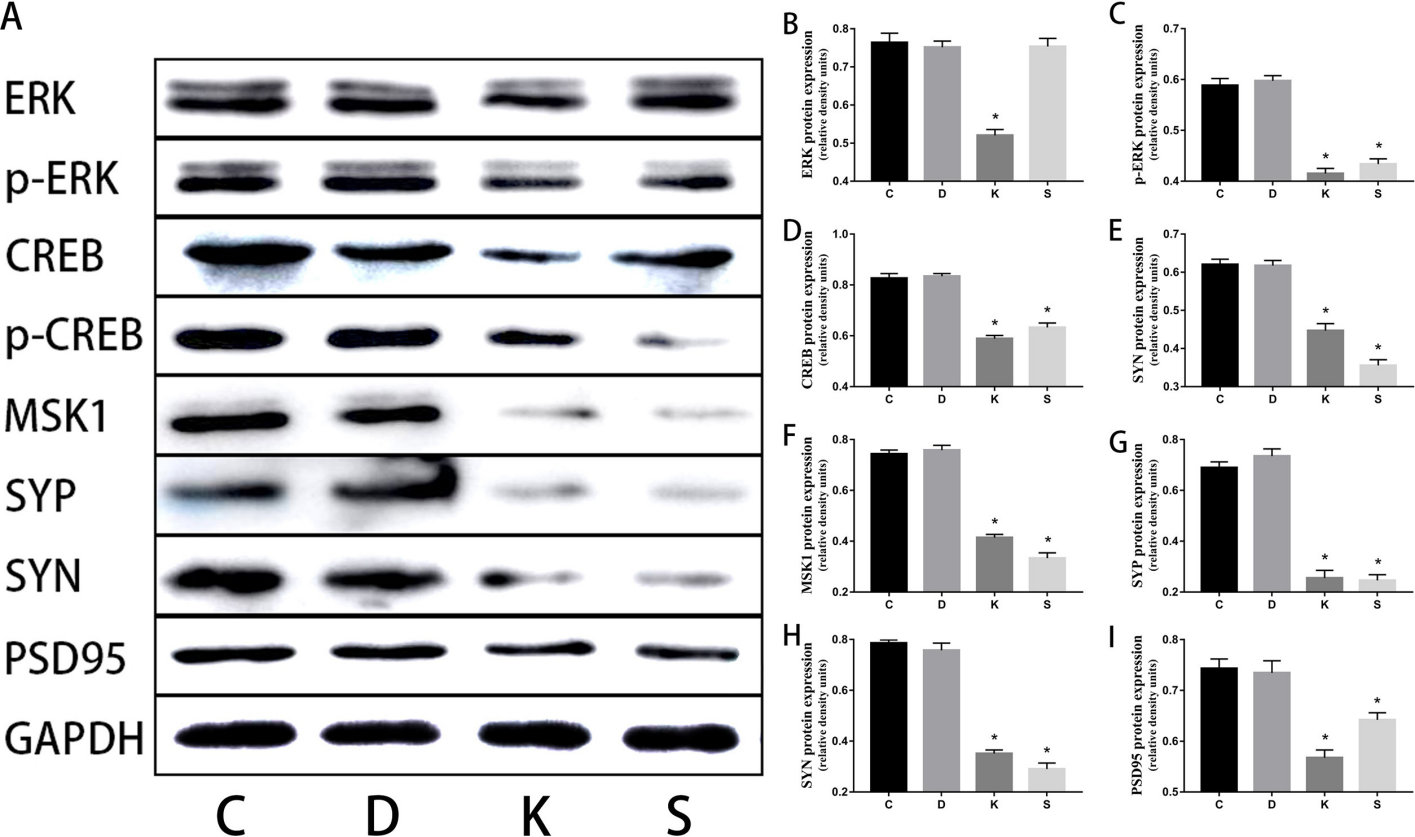
Western blot *in vitro* experiment Ketamine administered during the second trimester down-regulated ERK, p-ERK, MSK, CREB, p-CREB, SYP, SYN, and PSD95 in PC12 cells during *in vitro* experiments. SCH772984 is the inhibitor of the ERK protein. Through the contrast between S group and C group, a very good effect of the inhibitor is revealed. **(A)** Representative immunoblots for expression levels of the corresponding proteins. **(B–I**) Histograms that quantify the corresponding proteins normalized to GAPDH, in which the data are expressed as mean percentages ± SEM of control mean values. *p < 0.05

Three synaptic proteins in hippocampal specimens were visualized via SDS-PAGE and immunoblotting with corresponding antibodies for SYP, SYN, and PSD95. For proteins of hippocampal tissue, we found significantly decreased expressions of SYP, SYN, and PSD95 in K group relative to C group (*p* < 0.05, Figure 4G, 4H, 4I). Furthermore, we found the same trend in four cell groups (Figure 5G, 5H, 5I).

### Ketamine perturbed NGF protein and down-regulated ERK and CREB and their phosphorylation

Ketamine injection significantly reduced the levels of the ERK, p-ERK, MSK, CREB, p-CREB, and NGF proteins in the hippocampus compared to C group (*p* < 0.05, Figure 4A–4F). *In vitro* experiments using PC12 cells revealed an identical trend between C and K groups. In S group, the levels of these proteins were also significantly decreased (*p* < 0.05, Figure 5B–5F), except for ERK (Figure 5A).

## DISCUSSION

In this study, we found that the use of ketamine in pregnant rats at G14 might possibly affect both learning and memory of space and condition. The behavioral results were verified via Nissl and Golgi stainings. It has been reported that activation of the ERK-CREB signaling pathway may improve spatial learning and memory [19]. Therefore, we selected the area upstream and downstream of the key proteins of this pathway to measure expression levels using western blots. The results showed that the contents of NGF, ERK, CREB, and SYN were decreased. In this experiment, ketamine was shown to lead to changes in oxygen concentration, blood flow, and oxygen saturation. To exclude the effects of these factors, we cultured PC12 cells *in vitro* and added an inhibitor, thus validating the results of *in vivo* experiments. Two important aspects of neurocognitive function are learning and memory. Therefore, we speculate that the changes of ERK signaling pathway can affect the neurocognitive function.

Anesthesia is widely and increasingly used in the developing brain, making it a major health issue of vital interest. This has become a greater concern with the evidence that anesthesia and surgery may cause neuro-developmental disorders in children, while anesthetics are neurotoxic in young animals [22]. Recent studies suggested an association between anesthesia/surgery and neurocognitive impairment, and Sanders et al. suggested a causal relationship between anesthesia and neonatal brain injury [23]. However, the effects of anesthesia on the development of fetal brains, affecting postnatal memory and learning ability is controversial; studies reporting a lack of effects [24] and studies reporting permanent impairment [25] can all be found. However, these discrepancies may be due to differences in experimental methods, animals, drugs, and anesthetic durations. In fact, all general anesthetics pass the placental barrier and convincing evidence suggests that anesthetics, including ketamine, can potentially cause neuronal apoptosis in synaptogenesis as well as behavioral deficits in later life [26, 27]. According to behavioral test results, ketamine-treated rats have weaker spatial memory as manifested by increased latency during the Morris water maze test and conditional in the CS test; thus revealing that ketamine exposure can affect the learning and memory abilities of rat offspring, which is consistent with previous studies [27]. These findings clearly demonstrate that ketamine can cause neurocognitive functional impairment in offspring. These behavioral changes are unlikely associated with an indirect adverse effect of ketamine on pregnancy, as maternal physiological parameters during ketamine anesthesia were normal (Table 1). This further suggests that the offspring brain suffered from impaired via ketamine exposure. Moreover, under the conditions of this experiment, there were impairments of hippocampal-dependent learning and memory after ketamine exposure in the offspring. This suggestion is consistent with that of Peng et al. who suggested that ketamine impairs learning and memory ability possibly via inhibition of the ERK signal transduction pathway [28].

ERK is an important serine/threonine protein kinase, which is activated via phosphorylation on Thr202 and Tyr204 [29]. Its substrate includes histones, transcription factors, and other important intracellular kinases; substances which are important components of the cell, and play a central role in the formation of learning and memory. Once activated, ERK translocated from the cytoplasm to the nucleus, where it activated specific transcription factors that in turn induce gene expression. ERKs can activate MSK [30] and activated ERK can in turn activate CREB through MSK activation. ERK can directly phosphorylate the transcription factor CREB. Montarolo et al. reported for the first time the long-term effects of cAMP-CREB pathway facilitation activities in the memory process of Aplysia, and the authors introduced the first mechanism of learning and memory dependent gene expression [31]. There is considerable body of evidence to show that activation of ERK (p-ERK) in the hippocampus enhances memory via phosphorylation of the downstream transcription factor CREB on Ser residue 133 [32]. Low expression of CREB can lead to significant damage to the memory [33, 34], while high expression of CREB can result in significant enhancement of memory and an improvement on social cognitive function [35]. Results from Figure 8 have shown that ketamine injection significantly reduced the level of the ERK, p-ERK, MSK, CREB, and p-CREB proteins in the hippocampus. These results suggest that ketamine could inhibit the activation of CREB by suppressing the ERK/MSK signaling pathway. This hypothesis was verified by *in vitro* cell experiments. After inhibitor addition, the related proteins were inhibited; thus illustrating the important position of ERK in the signal pathway. These results are consistent with prior reports about the key role of ERK in the control of gene regulation required for neuronal plasticity and long-term memory in the central nervous system [36]. A recent report shows that CREB mediated the transcription of SYP via combination between p-CREB and the SYP promoter [37]. ERK is a family member of the mitogen-activated protein kinase pathway, which is strongly associated with synaptogenesis, learning, and memory [38].

**Figure 6:**
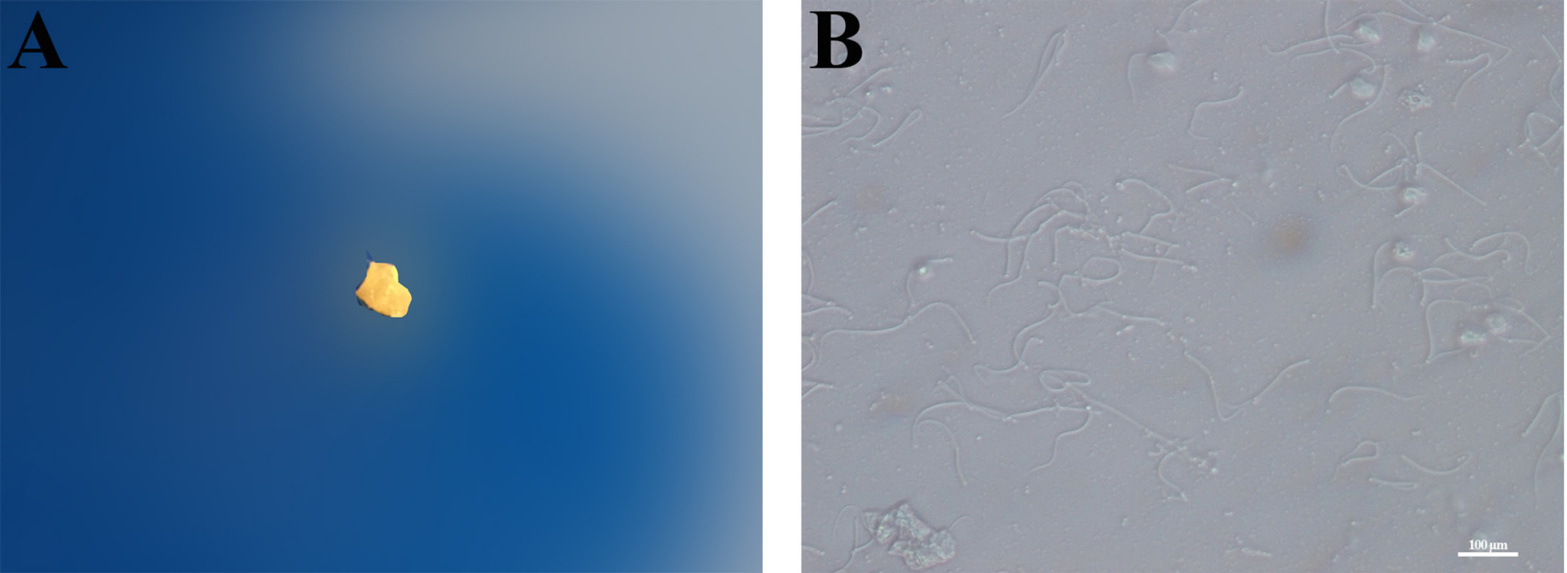
**(A**) Vaginal embolus; **(B**) Sperm in vaginal smear

**Figure 7:**
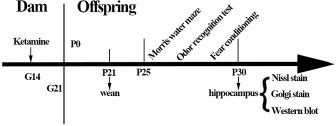
Flow chart of experimental protocols E = gestation day; P = postnatal day.

**Figure 8:**
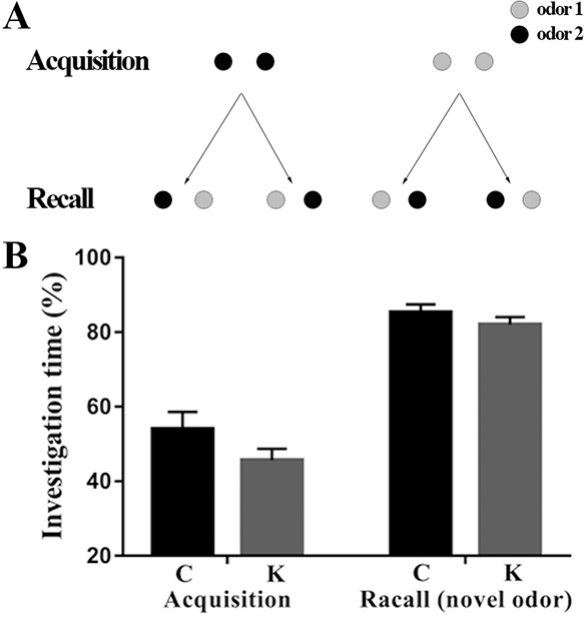
Odor recognition test **(A**) In a first acquisition test of 2 min duration, both holes were odorized with the same compound and were equally explored by the animals. In a recall test after a delay of 30 min, one hole was odorized with the previously presented odor during the acquisition test, while the other hole contained a new and unknown odorant compound. The placement of the familiar and new odors was randomized to avoid place preference bias. **(B**) Odor recognition test. Rats were subjected to the acquisition test (2 min) with either carvone or limonene present in both holes of the hole-board. The percent investigation times (vertical axis) of both holes were similar (open bars: “Acquisition,” Hole 1 and Hole 2). Immediately following the acquisition test, all animals received an i.p. injection of control (physiological saline) and were submitted to the recall test after a delay of 30 min. The bars (Recall-novel odor) represent the mean exploration time of the hole containing the new odor, expressed as the percentage of the total exploration time of both holes. At a delay of 30 minboth holes were equally explored, indicating that rats could no longer remember the familiar odor (one-sample *t-test*; *p* > 0.05).

The hippocampus is one of the key brain regions for long-term memory [8]. Furthermore, learning and memory are closely related to neuronal synapses of the hippocampus [39]. The results show that both learning and memory abilities of the offspring were impaired. Learning disabilities may not only be caused by neural regeneration, neurodevelopmental abnormalities, neuron migration, and neuronal apoptosis, but also by synapses [40]. Synapses are the contact point between neurons, which are the basic structure of many brain functions, particularly the ability of learning and memory [41]. In synaptic impairment, impaired synapses and neurotransmitters may further affect cognition. In this study, we assessed the levels of SYN and SYP proteins, two known markers for the presynaptic membrane, which are involved in the final steps of exocytosis and synaptic formation in hippocampal neurons [42]. The level of the PSD95 protein was also assessed, which is a known marker of the postsynaptic membrane that is particularly abundant in the hippocampus where it aids the maturation of connections and plasticity (dendritic arborization and axonal branching) [43]. Early exposure to general anesthesia has been reported to transiently reduce the SYN level in the developing rat brain [44]. Beurel et al. demonstrated that ketamine treatment decreased the hippocampal membrane PSD95 level [45]. These results suggest that anesthesia exerts a striking influence on the level of SYN and PSD95 proteins. In this study, the level of SYN, SYP, and PSD95 proteins were decreased, which indicated synaptic damage [46]. Synaptogenesis occurs after the birth of a rodent. Synaptic proteins play an important role in synaptic formation, remodeling, and function, thus providing synaptic stability during brain development. For neuronal differentiation and migration, neuronal communication is of key importance [47]. Immature neurons cannot establish meaningful synaptic connections during the key stage of brain development and die via apoptosis [48]. Nissl bodies are characteristic neuronal structures in the cytoplasm of dendritic cells and neurons, and they have been reported to be involved in protein production and are sensitive indicators of neuronal function [47]. Our results showed ketamine induced neuronal cell loss in the hippocampal CA1 and CA3 regions of offspring, which is consistent with previously published results [49], it might be related to vascular injury in brain [50, 51]. Furthermore, long-term neurocognitive function requires functional integrity of dendritic spines [52]. It has been reported, that sevoflurane can reduce the spinal density [53]. A recent study suggested that long-term inhalational anesthesia exposure leads to morphological changes of dendritic spines and synaptic loss [54]. In this study, our results showed decreased dendritic spine density in offspring caused by ketamine exposure. This indicated that synaptic function was disturbed and provided further evidence for a strong relationship between synapses and ketamine-induced neurocognitive dysfunction.

Neuronal synaptic growth requires NGF, which is a neurotrophic factor that plays a critical role in the differentiation, protection, and maintenance of cholinergic neurons [12], among which cholinergic neuronal dysfunction is associated with cognitive deficits [55]. NGF has a close relationship with the synapse, and can enhance synaptic communication, thus leading to an improvement of learning and memory [56]. Furthermore, the growth of axons and dendrites is critical for neuronal connectivity and alterations thereof, which may contribute to cognitive function [57]. Studies have demonstrated that NGF increased the levels of synaptophysin in cultured dorsal root ganglion neurons [58], thus promoting the formation of synaptic connections through the synthesis of synaptophysin [42]. It has been reported that chronic infusion of NGF can improve learning and memory functions through changes in hippocampus specific cells, including synaptogenesis and cell proliferation [16]. The NGF signaling pathway can stimulate ERK activity [16]. Then, ERK activation induces the activation of the CREB protein, stimulating CRE-dependent transcription and leading to NGF-induced improvement of learning and memory [59]. NGF induced glutamate release leads to CREB phosphorylation, which is necessary for synaptic activity and ultimately affects memory formation [60]. In this study, we found that ketamine resulted in a significant decrease in NGF, ERK, p-ERK, CREB, and p-CREB proteins *in vivo*. These results indicate that ketamine inhibited NGF-inducing activities, involving the phosphorylation of ERK and CREB in rats. Through the induction of NGF and inhibition of its downstream signaling, p-ERK and p-CREB, and the impaired synapse, ketamine ultimately attenuated neurocognitive function.

In summary, our results show that maternal exposure to ketamine during the second trimester of pregnancy resulted in long-term neurocognitive dysfunction of offspring rats, which was likely caused by an inhibition of the NGF-ERK-CREB pathway in the hippocampus.

## MATERIALS AND METHODS

### Animals

27 rats (351-387 g) were housed in polypropylene cages in a temperature and humidity chamber with a 12 h light/dark cycle, where rats had access to water and food *ad libitum*. All experimental procedures were performed according to the guidelines that have been approved by the Northeast Agricultural University, Harbin, China. All efforts were made to minimize the total number of utilized animals.

### Copulation

One randomly selected male rat and two female rats were put into a cage to enable free mating. On the second day, observations were made for the occurrence of a vaginal embolus in the cage and sperms on vaginal smear. If vaginal emboli (Figure 6A) or sperms were detected via vaginal smear (Figure 6B), the female rat was considered pregnant, and this day was marked as gestational day 0 (G0).

### Experimental protocols

18 dams were randomly divided into control (*n* = 9) and ketamine (*n* = 9) group. At G14 and since this time was equivalent to the second trimester of human pregnancy [61], the control group (C group) were left undisturbed in their home cages, while the ketamine group (K group) received a 200 mg/kg dose of ketamine (Gutian Pharmaceutical Co., Ltd. Fujian province, China) via continuous intravenous infusion with a pump (Sinomdt Co., Ltd, Shenzhen, China) via tail vein for 3 h. The total volume of katamine was less than 2 mL/100 g. This duration of ketamine infusion induced a sedative state between light anesthesia and deep sedation, evidenced by a lack of voluntary movement, decreased muscle tone, and minimal reaction to painful stimulation; however, without compromising any cardio respiratory functions [62]. Three rats per group were used for subsequent blood gas analysis. The latter was confirmed with blood gas analysis in a separate cohort (Nova Biomedical blood gas analyser, Massachusetts, USA, see the Results section). The core body temperature was measured via rectal probe and maintained between 36.5 and 37.5°C via servo-controlled infrared lamp and heating pad throughout the experiments (RWD life science Co. Ltd., Shenzhen, China). At the end of these infusions, dams were returned to their home cages after the righting reflex had recovered. Dams in each group were allowed to naturally give birth. The pups were allowed to grow up with their mothers until postnatal day (P) 25 (the day of birth was designated as P0) when pups were weaned (*n* = 108, 9/dam, P25-30) for behavioral tests. Subsequently, *ex vivo* brain samples were harvested at P30 for Nissl staining, Golgi staining, and western blotting (*n* = 36, 3/dam for each examination, see below). A schematic representation of experimental protocols is shown in Figure 7.

### Behavioral test

#### Morris water maze

To test hippocampal-dependent spatial cognition, the rats were trained in the standard Morris water maze [63]. The equipment consisted of a hidden white platform (10 cm in diameter and 1.5 cm below the water surface in the center of the SW quadrant). The morris water maze consisted of a black circular pool, filled with warm (23–25°C), opaque water. The area of the maze was divided into four equal-sized quadrants: NE, NW, SE, and SW. Each rat was successively placed into the water of each of the four quadrants and a maximum of 90 s was allowed to find the platform. If rats failed to find the platform within this time limit, they were guided to it, and then stayed there for 10 s. All trials were videotaped. Furthermore, a video track program allowed us to measure the time required to find the platform as well as other behavioral information of the spatial memory test (Coulbourn Instruments; USA). Therefore, we could use the output data for statistical analysis. On the sixth day, the platform was removed to conduct a probe trial, testing the ability of the rats to identify the specific location that previously contained the hidden platform. The rats were then allowed to explore for 90 s and the number and time of entries into the platform quadrant zone were recorded. All rats were dried after completing each test.

### Odor recognition test

Olfaction is a highly developed sense in rodents. Olfactory discrimination tasks are excellent tests of learning and memory in rats and mice. For the present study, an odorized hole-board was designed and constructed specifically for mice [64]. This equipment had two holes (3-4 cm in diameter with a depth of 4.5-5 cm). At the bottom of the holes, a polypropylene swab was embedded in a plastic mesh and covered with wood shavings and contained 20 μL of diluted odors (1:10). In the acquisition test, one of these two odors (either carvone or limonene) was simultaneously present in both holes of the hole-board (Figure 8A) for a 2 min period. The recall test consisted of a second 2 min session in which one of both holes contained the previously presented odor, while the other hole contained the new odor (Figure 8A). The delay between acquisition and recall tests was 30 min. Furthermore, in both tests, the exploration time of rats at each hole was measured. If the rats spent less time exploring the hole containing the familiar odor than the time spent exploring the hole containing the new odor, they were considered to have remembered the familiar odor. If they take approximately the same time, this was considered to indicate that rats had forgotten the familiar odor. Both odors were alternatively used during the tests and randomly presented in each of the two holes to avoid place preference bias.

### Fear conditioning

**Figure 9:**
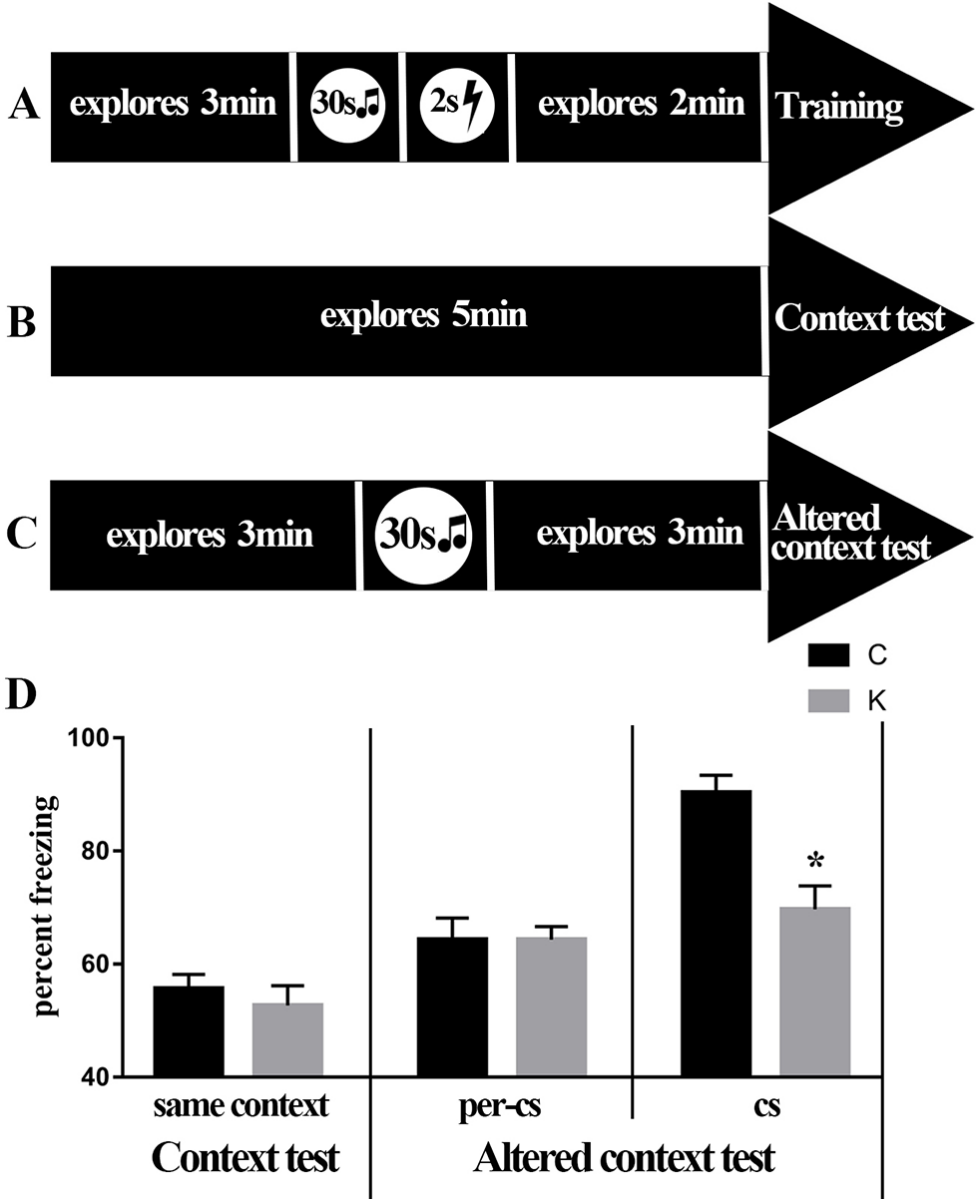
Fear conditioning **(A–C)** Flow chart of the fear conditioning test. **(D)** The histogram shows that no significant difference was detected in the context test and the pre-cs test (*p* > 0.05) and a significant difference was detected in the cs test (*p* < 0.05).

Fear conditioning is often chosen as a second independent learning and memory task that complements the Morris water task. This test consisted of three parts, training, context, and altered context test, all occurring 24 h apart. during training phase, rats were placed in the chamber for 3 min for habituation and then exposed them to a mild foot-shock paired with an auditory cue (tone, 30 s, 80 dB, 2 500 Hz; foot-shock, 2 s, 1 mA; foot-shock was delivered during the last 2 s of the tone presentation) (Figure 9A). A contextual test was performed in the same condition chamber for 5 min without any stimulation (Figure 9B) and the freezing time was recorded. An altered context test consisted of two parts, a pre-conditioning-stimulus (pre-CS) test and a conditioning stimulus (CS) test. In the Pre-CS test, rats were placed into a completely altered chamber, where they remained for 3 min. Then, during the CS test, the same training tone was delivered for further 3 min (Figure 9C) and the freezing time was scored. Freezing was defined as a completely immobile posture except for breathing. Therefore, associative learning and memory were assessed via length of freezing time.

### Nissl staining

After the behavioral test, rats were perfused with 0.9% saline solution, followed by 4% PFA in 0.1 Mphosphate buffer at pH 7.4. Brains were then removed and fixed for 6–8 h at 4°C. Following paraffin embedding, three coronal slices were collected from each rat, containing the dorsal hippocampus at 5 μm thickness. Then, toluidine blue staining (Nissl staining) was performed. Representative photomicrographs of the pyramidal cell layer of CA1 and the CA3 region were microscopically captured. Quantitative analysis of the ratios of viable neurons was processed via Image-Pro Plus (Leica DMLB) software at 400 × magnification. An average of three Nissl-stained sections was calculated to yield one single parameter per rat. All microscopic analyses were conducted by an observer blinded to the groups.

### Golgi staining

Golgi staining to obtain the hippocampal dendritic spine density was conducted with the FD Rapid Golgi Stain TMKit (FDNeuro Technologies Inc., Ellicott City, MD), following the manufacturer's instructions. Coronal tissue sections of 150 μm thicknesses were sliced at room temperature using a vibratome (Leica VT1200S, Germany). After slides were dehydrated with a gradient of 50%, 75%, 95%, to 100% ethanol and cleared in xylene, we prepared the specimens, using slide coverslips and sealed them with Permount. The slides were then viewed in detail with a light microscope (Leica DFC420, Germany). We analyzed the stained spine, using techniques similar to those described in other studies [65, 66]. Six well-impregnated pyramidal neurons that were clearly distinguishable from others in each hippocampus were analyzed (20 × objective lens). Five segments of 10 μm (or longer) of apical dendrites each were randomly selected from each pyramidal neuron for closer inspection (via 100 × oil immersion lens) to quantify the spinal density. Spinal density of secondary apical dendrites was analyzed at proximal segments, emerging at more than 50 μm from the soma of the hippocampal CA1 and CA3 neurons. All of these spines were required to have a clearly distinguishable base or origin and had to be isolated from neighboring dendrites. Spinal density was calculated per 10 μm of dendritic length. The open-source ImageJ1.48 r Java image-viewing and image-processing software (Wayne Rasband, NIH, USA) was utilized to calibrate the scale and enlarge the segment of the spines. All analyses were completed by an investigator blinded to experimental conditions.

### Cell culture and treatment

PC12 cells were obtained from the College of Pharmacy of the Sun Yat-Sen University (Guangzhou, China). Cells were seeded in 25 cm^2^ flasks at a density of 1×10^5^ cells and maintained in DMEM, supplemented with 100 U/ml penicillin (Beyotime Biotechnology), 100 U/ml streptomycin (Beyotime Biotechnology), and 10% fetal bovine serum (FBS, biological industries) at 37 °C in a humidified atmosphere of 95% air and 5% CO_2_. 24 hours prior to the start of experiments, NGF was added to induce synaptic production in PC12 cells. The cells were randomized into 4 groups: the control group (C group), the ketamine group (0.6 μg/mL, K group), the DMSO group (D group), and the SCH772984 group (10 μmol/L, S group). The cells of these four groups were subcultured for 24 h in the same conditions than prior to incubation with these treatments. After 3 h of culture, the extracted protein was used for western blotting.

### Western blot

Western blot analysis was performed as previously described [67]. In brief, neurons were harvested in a cell lysis buffer. Hippocampal fragments were isolated after behavioral test and stored in a −80°C freezer until further use. At the time of processing, tissue lysis buffer (containing protease inhibitors, 50 mM Tris-HCl, at a pH of 7.6) was added to the samples on ice; then, the tissue was homogenised via ultrasonification (Ningbo scientz biotechnology Co. Ltd., Ningbo, China) and centrifuged at 12 000 g for 10 min at 4°C. The protein concentration in the supernatant was measured with a BCA assay kit (Beyotime Institute of Biotechnology, China). Then, the supernatants were mixed with gel loading buffer (50 mM Tris–HCl, 10% SDS, 10% glycerol, 10% 2-mercaptoethanol, 2 mg/mL bromophenol blue) at a ratio of 1:1 and boiled for 5 min. Then, subjected to SDS-PAGE (10% acrylamide gels for GAPDH ERK, p-ERK, CREB, p-CREB, MSK1, SYP, SYN, PSD95, and 15% acrylamide gels for NGF, 120 V, 70 min). The separated proteins were transferred onto nitrocellulose (NC) membranes and incubated with rabbit anti- GAPDH, NGF, ERK, p-ERK, CREB, p-CREB, MSK1, SYP, SYN, and PSD95 (1:600, EnoGene, Nanjing, China) overnight at a temperature of 4 °C. The membrane was exposed to either rabbit or mouse secondary antibodies (1:2 000, EnoGene, Nanjing, China) for 1.5 h at room temperature. The blots were visualized via Scanmaker 3836 (Microtek Technology co., LTD, Shanghai, China) and quantified with the Quantity One software (Bio-Rad Laboratories, Inc., USA). Expressions of NGF, ERK, p-ERK, CREB, p-CREB, MSK1, SYP, SYN, and PSD95 were determined by calculating their density ratio to the GAPDH band, and then normalized to the control group.

### Statistical analysis

All statistical parameters were calculated using the GraphPad Prism 7.0 software package. Values are expressed as the mean ± S.D. Behavioral test, Nissl staining, Golgi staining, and Western blots *in vivo* were evaluated with the Student's *t-test*. Western blots *in vitro* were analyzed via one-way ANOVA analysis followed by Tukey's post hoc test. Differences with a *p-value* below 0.05 were considered statistically significant.
